# What is the future of the Brazilian Public Health System?

**DOI:** 10.31744/einstein_journal/2018ED4811

**Published:** 2018-11-29

**Authors:** Jacyr Pasternak

**Affiliations:** 1 Hospital Israelita Albert Einstein, São Paulo, SP Brazil

We are the country with the largest population on the planet that has a comprehensive healthcare service, with no “out-of-pocket” payment by end users.^(^ ^1^ ^)^ It was clearly inspired by the British model of the National Health Service. The Brazilian system has some aspects that work very well: our vaccination program is excellent and provides more types of vaccines than richer countries.^(^ ^2^ ^)^ Our healthcare system for HIV-infected individuals is outstanding. Nevertheless, the Brazilian Unified Health System (SUS - ***Sistema Único de Saúde)*** suffers from a chronic shortage of financing^(^ ^3^ ^)^ and lack of coordination among the various authorities of the Republic responsible for its management. The system is extremely fragmented, and management is often given to people appointed for political commitments, and not by their merit. Coordination among the various levels of managers is faulty, and investment in the system has dropped constantly. Brazil does not invest poorly in health (approximately 9% of its Gross Domestic Product) - in that, 47% of expenses are paid with public funds and the remaining (53%) comes from the private sector. Countries with structures similar to that of SUS, such as the United Kingdom (which invests 83.2% in the public health system), Canada (71.1%), Italy (77.6%), and Holland (84.8), show how small is our portion of expenditure on public health, compared to what is spent in the United States, which invests 48.5% of funds available in public healthcare systems, such as Medicare.^(^ ^3^ ^)^

One particular aspect of SUS is the preferential use of its resources by people who are far from the Brazilian economic base. Costly medications or drugs unavailable in the country are regularly provided to people with knowledge and expertise, who know how to request them by going through the Judiciary power, whereas more socially deprived people simply get lost in the attics of the system. As stated by one of our professors who also served as the Minister of Health, Dr. Adib Jatene, “the problem of the poor is not only being poor, but also not knowing anyone with influence.” Similarly, people who are better educated and who have richer networking know where the resources are more readily available, and where the best trained physicians are located.

With the economic crisis we are going through, approximately 3 million people lost access to their health insurance plans and had to resort to SUS. Equally, more complex or more expensive procedures go through the supplementary services. From there they are referred to SUS, which deals with high-complexity cases, while the supplementary services deal with less complex and less severe patients. It is not even possible to say that this is wrong, because according to the laws of the country, all Brazilians - both rich and poor - have the right to an unrestricted access to the healthcare programs, including diagnosis, treatment, medications, and rehabilitation. The legislators forgot, however, to provide the resources necessary for all this. As the population ages, and new drugs - increasingly more expensive - are launched, besides new types of diagnostic tests, it seems evident that, if the current method is maintained, SUS will not be capable of providing what is mandatory by law. *Mutatis mutandi:* this reproduces the same picture of our welfare, which clearly will soon become unworkable.

We do not have the courage to propose a solution, if it exists. To decrease the extreme fragmentation of the system, improve management, invest more in the initial care of users, and promote health before disease are all evident needs, but they alone will not solve all the problems. To reduce bureaucracy as much as possible, retain physicians in the public system with a decent career, and coordinate several municipalities so that they act jointly (this is valid primarily for the many municipalities that are economically not viable and live with only the transfers from the State and Federal governments) are essential. To electronically improve the information of all users is an ancient need, which until now, has not been attended to.^(^ ^4^ ^)^

One final aspect: SUS is basically paternalist and treats its users as children, with all of the privileges and few responsibilities.

We would suggest that it treats everyone as adults, that is, each individual is responsible for their health. Health is not only the duty of the State, it is also an obligation of each person. It is very comfortable to leave everything in the State's hands, and certainly, this attitude has everything going against it, as history has proved…

## References

[1] Heath I (2018). Back to the future: aspects of the NHS that should never change-an essay by Iona Heath. BMJ.

[2] (2015). Sato API National Immunization Program: Computerized System as a tool for new challenges. Rev Saude Publica.

[3] Massuda A, Hone T, Leles FA, de Castro MC, Atun R (2018). The Brazilian healthsystem at crossroads: progress, crisis and resilience. BMJ Glob Health.

[4] Mendes EV (2013). 25 anos do Sistema Único de Saúde: resultados e desafios. Estudos Avançados.

